# Determination of the Presence of Diphtheria Toxin in the Myocardial Tissue of Rabbits and a Female Subject by Using an Immunofluorescent Antibody Method

**DOI:** 10.14740/jocmr2142w

**Published:** 2015-04-08

**Authors:** Mehmet Ceyhan, Yasemin Ozsurekci, Merve M. Aydin, Kamil Can Akcali, Beril Talim, Melda Celik, Eda Karadag Oncel, Venhar Gurbuz, Ahmet Emre Aycan, Ilyas Onbasilar, Turan Buzgan

**Affiliations:** a Department of Pediatrics, Division of Infectious Diseases, Hacettepe University Faculty of Medicine, Ankara, Turkey; b Department of Molecular Biology and Genetics, Bilkent University, Ankara, Turkey; c Department of Pediatrics, Pathology Unit, Hacettepe University Faculty of Medicine, Ankara, Turkey; d Laboratory Animal Breeding and Research Unit, Hacettepe University Faculty of Medicine, Ankara, Turkey; e Turkish Public Health Institute, Turkey; f These authors contributed equally to this work and shared first authorship.

**Keywords:** Diphtheria, Diphtheria antibody, Diagnosis, Animal model

## Abstract

**Background:**

Clinical diagnosis of diphtheria is often difficult, in particular in countries where the disease is rarely observed, such as Turkey. In 2011, after 12 years of no recorded diphtheria cases in Turkey, a 34-year-old woman was diagnosed with diphtheria; she later died of myocarditis. In this study, we aimed to demonstrate the diagnostic potential of an immunofluorescent antibody method to determine the presence of diphtheria toxin (DT) in the myocardial cells of DT-injected rabbits and the female subject.

**Methods:**

We randomly divided rabbits into two groups: a control group and a DT-injected group. Diphtheria intoxication was simulated in the rabbits by intravenous injection of DT. The myocardium of the rabbits and the female subject were harvested for histopathologic and immunofluorescence examination. A mouse monoclonal anti-DT antibody was used for the immunofluorescent antibody method.

**Results:**

The presence of DT in the myocardial cells of both the rabbits and the female subject was visualized using the immunofluorescent method.

**Conclusions:**

Laboratory diagnosis of diphtheria is challenging because of non-toxigenic *C. diphtheriae* strains and/or the dysfunction of DT. However, visualizing the presence of DT in the myocardial tissue may act as an indicator of biologically active DT. We validated that an immunofluorescent method, which utilizes a monoclonal anti-DT (A-subunit specific) antibody, is a useful diagnostic tool to determine the presence of DT in the myocardium of rabbits and human.

## Introduction

Diphtheria is an acute, communicable disease caused by exotoxin-producing *Corynebacterium diphtheriae*. The disease is generally characterized by local growth of the bacterium in the pharynx with pseudomembrane formation or, less commonly, in the stomach or lungs; systemic dissemination of the toxin then invokes lesions in distant organs [1]. Diphtheria toxin (DT), the main virulence factor produced by the causative organism *C. diphtheriae*, is an extremely potent bacterial toxin with a minimal lethal dose [2-4]. The 50% lethal dose per kilogram (LD_50_) of DT for humans was about 100 ng/kg or less [5]. Diphtheriae toxin does not have a specific target organ, but the myocardium and peripheral nerves are most affected sites [6]. Myocardial damage is a well-known and sometimes fatal complication of diphtheria [7, 8].

Experimental investigations have shown that DT alters protein and fatty acid metabolism in the cardiac tissue [7, 8]. Therefore, the most noteworthy tests in the microbiological diagnosis of diphtheria include rapid and accurate detection of the potent and lethal exotoxin from a suspected clinical isolate. Toxigenicity is currently determined in most laboratories by the Elek immunoprecipitation test [9], a method prone to misinterpretation, in particular in laboratories where it is performed infrequently. The clarity and accuracy of the test depend on the constituents of the medium, the concentration of antitoxin, and the use of appropriate control strains [10]. The limitations of current methods are their inability to differentiate between the biologically active and inactive forms of the toxin. Genotyping methods, based upon PCR, offer many advantages over phenotyping techniques; they are rapid, simple, and easy to interpret, and PCR facilities are becoming increasingly available in many laboratories. However, these methods do not provide information on the ability of the organism to express the fully functional DT [11].

In the 2011, after 12 years of no recorded clinical cases of diphtheria in Turkey, a 34-year-old woman who developed sore throat, fever, and dysphagia was diagnosed with diphtheria. Based on her medical history, the patient had not been vaccinated with tetanus and DT during childhood. The clinical diagnosis of diphtheria was made by Elek and PCR tests. The patient was treated with diphtheria antitoxin and intravenous antibiotics. However, she was later transported to the intensive care unit with a diagnosis of myocarditis. Cardiac findings of the patient including electrocardiogram and cardiac enzymes were reported previously [12]. She died 10 days after the beginning of the treatment. Postmortem heart necropsy material was obtained. Because of the virulent nature of DT, throat swab samples were taken from her relatives as well as health care personnel who were in contact with the patient. All the relatives and health care personnel were administered the appropriate antibiotic treatment and were vaccinated according to age groups. *Corynebacterium diphtheria variant gravis* was isolated from the patient and one child of the patient. The classmates were swabbed after the child was determined to be positive and *C. diphtheria variant gravis* was isolated from four of the child’s classmates. These children and their parents were treated and vaccinated according to age groups. At the end of 1 month after the initial diagnosis of the first patient, there were no new clinically diagnosed cases.

The diagnosis and subsequent death of the patient of acute diphtheria provided an opportunity to study the histopathologic changes induced by DT in the heart. The ideal test for use in the diagnostic laboratory must be shown to correlate with the biological activity of DT. Visualizing the presence of DT in myocardial tissue may be an indicator for biologically active DT. In the present study, an immunofluorescent antibody method was used to confirm the presence of DT in the myocardial cells of the patient and in an experimental setting in DT-injected rabbits.

## Materials and Methods

### Animals and experimental design

This study was conducted at the Hacettepe University Faculty of Medicine, Pediatric Infectious Diseases Unit with the approval by the Hacettepe University Institutional Ethics Committee for experimental animal studies (B.30.2.HAC.0.05.06.00/20) and following the Guidelines for the Care and Use of Laboratory Animals of the US National Institutes of Health (Washington, DC).

A rabbit model was designed to study the presence of DT in the myocardial tissue because rabbits are one of the few animals that are not resistant to DT [13]. We housed New Zealand albino rabbits and provided them with regular laboratory chow and water. Rabbits (n = 9) were divided into two groups. Rabbits in group 1 (control group; n = 3) were not exposed to DT. Rabbits in group 2 (n = 6) were exposed to DT. We used DT from *C. diphtheriae* lyophilized powder (D0564; Sigma, Taufkirchen, Germany) to infect the rabbits. The LD_50_ of DT for sensitive species including rabbits was about 0.1 µg/kg, irrespective of injection route. The dose was expressed as µg of toxin causing death within 7 days/kg of animal body weight [13]. Diphtheria intoxication was simulated in the rabbits by intravenous injection of 0.4 µg/kg DT once a day until death in group 2. The dose was determined as four times the lethal dose to ease the suffering of the animals and to ensure death within 3 days. All the rabbits in group 2 died within 72 h.

### Tissue preparation

#### Human tissue

Necropsy material was obtained from the walls of the cardiac chambers approximately 6 h after death of the patient. Some of the heart tissue was fixed in 10% buffered formalin and embedded in paraffin for histopathologic evaluation, while the rest was frozen in isopentane cooled in liquid nitrogen and stored at -80 °C for histochemical and immunofluorescent examination.

Written informed consent was obtained from the patient’s parents and husband for the necropsy, publication of the patient’s reports, and any accompanying images.

#### Animal tissue

After the death of each rabbit, the chests were opened, and the hearts were dissected. The tissue specimens were flushed with cold saline solution, and small portions of the cardiac tissue were fixed in 10% buffered formalin, processed for paraffin sections, and stained with hematoxylin-eosin (H&E) for histopathologic evaluation. The remaining portion of the heart tissue was stored at -80 °C for immunofluorescent examination.

### Histopathologic studies

One slide from each specimen was stained with H&E for histological assessment. Oil-Red-O stain was done to show lipid accumulation in frozen heart muscle.

### Immunofluorescence staining for frozen tissue

Sections (5 µm) of frozen tissue samples were fixed with 4% paraformaldehyde for 30 min at room temperature. After washing with 1 × PBS-T (0.1%) for 5 min, the sections were incubated for 1 h with 1% BSA blocking solution that contained 0.25% Tween 20. After the blocking step, a primary antibody against the alpha subunit of DT (RayBiotech, Norcross, GA) was used at a dilution of 1:25 for the human samples and 1:50 for the rabbit samples. The sections were incubated overnight at 4 °C. After washing with 1 × PBS-T (0.1%) for 5 min, the tissues were incubated with FITC-labeled anti-mouse IgG (Sigma, St. Louis, MO, USA) at a dilution of 1:200 in blocking solution at room temperature for 1 h. After washing with 1 × PBS-T (0.1%), the specimens were mounted using UltraCruz^™^ (Santa Cruz, California, USA) mounting medium containing DAPI and examined under the fluorescent microscope (Leica TCS/SP5, Japan). The excitation wavelengths for FITC and DAPI were set at 490 nm and 359 nm, respectively.

## Results

### Histopathological examination of the human myocardium

**Figure 1:**
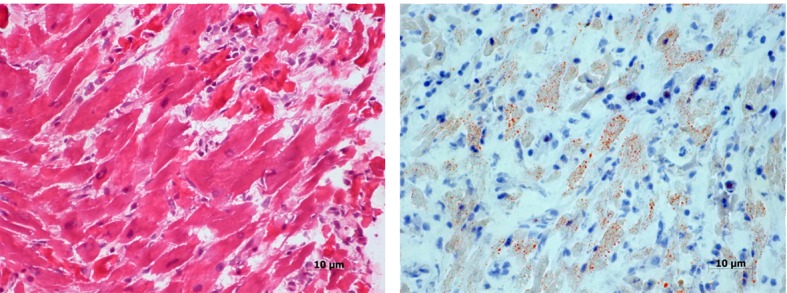
Section from the patient’s myocardium showing mononuclear inflammatory cell infiltration, degeneration and necrosis of some cardiac myocytes (A). Oil-Red-O stain reveals increased lipid in some fibers (B).

Areas of inflammatory infiltration, predominantly by mononuclear cells and few scattered neutrophil leucocytes, degeneration and necrosis of cardiac myocytes and interstitial edema were the main pathological features (Fig. 1A). Lipid content of some fibers was mildly increased (Fig. 1B).

### Immunofluorescent staining of the human myocardium

**Figure 2:**
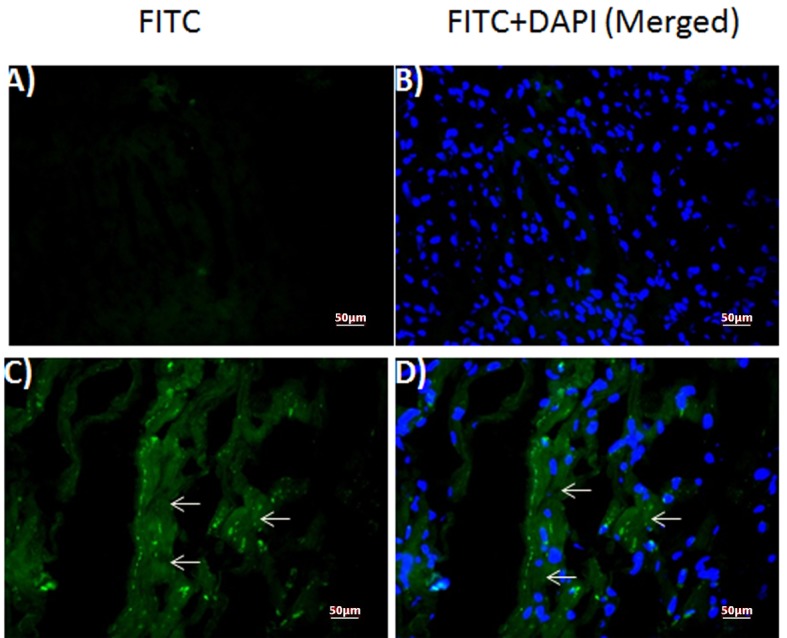
Expression of diptheria toxin in human myocardium. Fluorescence photomicrographs of (A, B) normal control and (C, D) patient myocardium sections. (A, C) diptheria toxin-FITC (B, D) merged with DAPI. Arrows: FITC-positive diptheria toxin.

Immunofluorescent staining against DT was performed on healthy control (Fig. 2A, B) and patient’s myocardium (Fig. 2C, D). Our results revealed diffuse staining in the frozen sections obtained from the patient (Fig. 2C, D), but not in those obtained from the healthy control (Fig. 2A, B). Because DAPI was used as a counter-stain to reveal the nuclei, merged images of the same sections with DAPI and FITC filter (Fig. 2B, D) localized the DT staining to the cytoplasm in the patient sample (Fig. 2D).

### Histopathological examination of the rabbit myocardium

No significant histologic (H&E staining) features were observed in case of rabbit myocardium.

### Immunofluorescent staining of the rabbit myocardium

**Figure 3:**
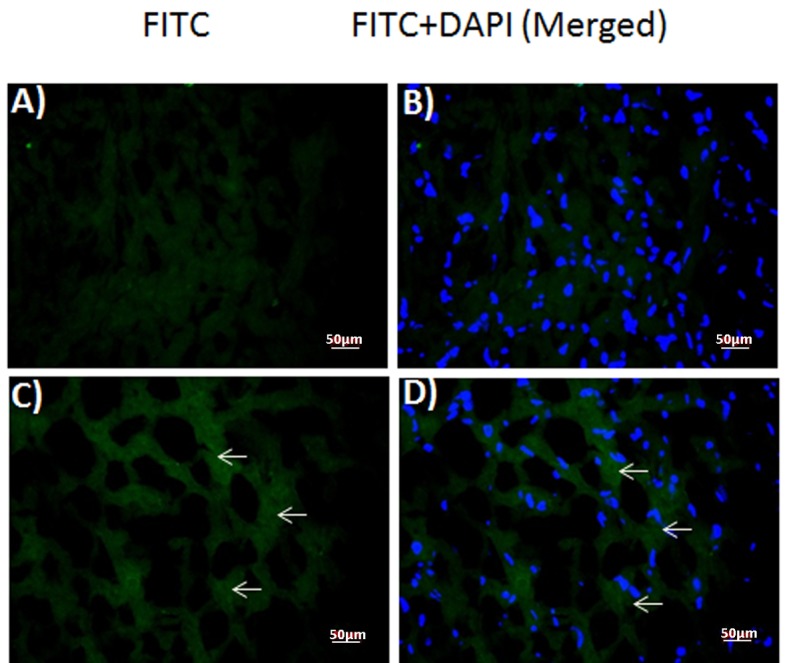
Expression of diptheria toxin in rabbit myocardium. Fluorescence photomicrographs of (A, B) control and (C, D) toxin-injected (1.2 µg) rabbit myocardium sections. (A, C) diptheria toxin-FITC (B, D) merged with DAPI. Arrows: FITC-positive diptheria toxin.

Immunofluorescent staining against DT was performed in the frozen sections of normal control (Fig. 3A, B) and DT (1.2 µg) injected rabbit myocardium (Fig. 3C, D). Samples from toxin-injected animals stained positively (Fig. 3C, D), whereas those from control animals did not (Fig. 3A, B). Merged images of the same sections with DAPI and FITC filter (Fig. 3B-D) localized toxin staining to the cytoplasm of the injected animals’ samples (Fig. 3D), similar to the human patient sample.

## Discussion

By the beginning of the 1980s, available evidence suggested that diphtheria persisted as a health concern [14]. Diphtheria remains a potentially fatal disease clinically presenting with membranous pharyngitis, often with complications of myocarditis and less commonly neuritis and respiratory co-infections such as pneumonia or bronchitis [15, 16]. Clinical diagnosis of diphtheria is often difficult, in particular in countries where the disease is rarely seen, such as Turkey. Prior to 2011, a clinical case of diphtheria in Turkey had not been diagnosed for 12 years. At present, most physicians have little experience in diagnosing and treating diphtheria. The diagnosis would normally precede microbiological diagnosis. The microbiological diagnosis of diphtheria has traditionally relied upon assays that are either technically demanding or greatly prone to misinterpretation [11]. The ideal test for use in the diagnostic laboratory must be shown to correlate with the biological activity of DT. Therefore, in this study, we aimed to demonstrate the diagnostic potential of an immunofluorescent staining method to determine the presence of DT in the myocardial cells of rabbit and human. We report the following major findings: 1) the presence of DT was revealed in the myocardium by using a mouse monoclonal anti-DT antibody, which has been recently used as a relatively reasonable diagnostic tool; 2) the level of immunity declines in late childhood and adolescence depending on the immunization schedule and the remaining reservoir of *C. diphtheriae* in the population, which may lead to gaps in the immunity of the adults, and diphtheria outbreaks may occur in subgroups of susceptible individuals despite widespread childhood vaccination, as observed in Turkey; and 3) a rabbit model for the study of diphtheria might enable us to understand the underlying mechanisms and diagnostic modalities of diphtheria-related diseases.

The Elek immunoprecipitation test is still used in many laboratories worldwide; however, this test is prone to misinterpretation, in particular when it is performed infrequently [10]. Therefore, an approach to strengthen the diagnosis of diphtheria with accurate methods is required, despite the positive aspects of Elek test. Hallas et al used a monoclonal antibody in an enzyme-linked immunosorbent assay for the detection of DT [17]; occasional false-positive results due to the nonspecific binding of the monoclonal antibody to the defective toxin were documented in this study. In another study, immunoblotting with a monoclonal antibody specific for subunit A of the toxin was used to assess the presence of the toxin in whole-cell lysates of pathogenic strains of *Corynebacterium* species. Efstratiou et al [11] reported complete concordance between the immunoblot detection of the subunit A domain and toxigenicity, as determined by functional assays. The limitation of current immunologic assays is the inability to differentiate between the biologically active and inactive forms of the toxin. Their specificity and sensitivity depend on the reactivity profiles of the antibodies used. Polyclonal antibodies directed at multiple epitopes on the toxin molecule are unlikely to differentiate between the intact active toxin and the biologically inactive toxin, whereas a panel of well-defined monoclonal or anti-peptide antibodies specific for DT functional domains might be more suitable for toxin detection [9]. In this study, FITC-conjugated goat anti-DT polyclonal antibody was used initially for the immunofluorescence studies, and it was ineffective. However, the presence of DT was revealed using the mouse monoclonal anti-DT antibody. Our findings suggest that the immunofluorescent technique that employed the monoclonal anti-DT (A-subunit specific) is potentially useful as a diagnostic tool to demonstrate the presence of DT in the myocardium. Our finding is consistent with Burch et al [18], they reported that the toxin demonstrated by the immunofluorescent technique was patchy in distribution and was often located within easily identifiable myocardial fibers in a pediatric case previously. This may also be an indirect indicator of the biological activity of the toxin.

Despite the widespread use of immunization, diphtheria remains endemic in several regions including Africa, India, Bangladesh, Vietnam, and Brazil [19-23]. Sporadic cases still occur, and the majority of diphtheria cases originate from endemic areas [15, 24]. The causes for the re-emergence of an epidemic in countries where immunization programs had nearly eliminated diphtheria are not fully understood, but they are thought to include the introduction of toxigenic *C. diphtheriae* strains of a new biotype into the general population, in addition to the low coverage of the diphtheria vaccine among children and the large gap of immunity among adults [25] as observed in case of the patient in the present study. The widespread availability of DT led to a marked decrease in the incidence of diphtheria and in the circulation of toxigenic *C. diphtheriae*, resulting in less natural boosting of antibody levels [14, 26, 27]. In Turkey, DT has been implemented in the immunization program at 1968. Since 2008, combined diphtheria, acellular pertussis, tetanus, *Haemophilus influenzae* type b and poliomyelitis (DTaP-IPV-Hib) is administered at 2, 4, 6 and 18 months of age. A booster dose, diphtheria-tetanus (DT) vaccine is administered at 7 years of age (at the first class of primary school). In 1997, because of diphtheria outbreaks in the neighboring countries such as Soviet Union [19], a second booster dose of tetanus-diphtheria (dT) vaccine was introduced at 12 - 15 years of age [27]. After 20 years of age, the protection rate was gradually decreased by age in some reports from Turkey [27, 28]. Additionally, low protection rates among females were reported in many studies [29, 30] as observed in Turkey [27, 31]. Although health authorities recommend a booster dose of Td every 10 years, there is no comprehensive Td vaccination for adults in Turkey [26, 32]. According to the records of the Turkish Ministry of Health, the vaccination coverage of the area, where the patient was born, was 16% during the 1980s. In Turkey, the cause of the re-emergence of diphtheria may be attributed to this large gap of immunity among adults.

In addition, *C. diphtheria variant gravis*, a biotype currently found circulating within Europe where diphtheria remains epidemic, was isolated from the patient, one child of the patient and four of the child’s classmates. The emergence of the epidemic clone of toxigenic *C. diphtheria variant gravis* was first documented in 1987 and accounted for an increasing proportion of the strains isolated from cases in sentinel areas as the epidemic progressed [33]. Those cases reinforce the potential susceptibility of Turkish adults to diphtheria in the vaccine era. When contemplating the epidemics that were recorded over the last decades in Europe [34] and Russia [35-38] understanding the geostrategic importance of Turkey, located at the crossroads of Europe and Asia, makes it a country of substantial consequence for *C. diphtheriae* infection and epidemics.

This study has several limitations. First, the developed monoclonal-DT antibody was proposed as a diagnostic tool in the manuscript; however, the test based on myocardium tissue in human is likely to be performed only during autopsy and it is unlikely to be a diagnostic test in clinical practice. However, the importance of the demonstration of the toxin *in situ* within the myocardium cannot be underestimated. Therefore, further animal and/or human studies are needed to understand the benefits of the work presented in clinical settings. Perhaps, nasopharyngeal or tonsillar tissue may be the next target to show the presence of DT. Second, we could not perform further analyses, because of technical and economic reasons, to calculate the amount of DT in the obtained samples and sequencing analyses that might allow us to understand the new biotypes of *Corynebacterium*.

In conclusion, laboratory diagnosis of the diphtheria can be problematic because of non-toxigenic *C. diphtheriae* strains and/or the dysfunction of DT. Given the immense public health implications associated with the isolation of a toxigenic strain of *C. diphtheria*, the delay between the time of isolation of a suspicious organism and the time that the results of toxigenicity tests are available can provoke great anxiety among laboratory staff, clinicians, and public health officials. The procedures for undertaking toxigenicity tests in a microbiology laboratory vary and depend on the facilities and the resources available, the expertise of the personnel, and the availability of a diphtheria reference laboratory for that country [11]. We believe that demonstrating the presence of DT in tissue will be an indirect indicator of its functional capacity.
